# Roscovitine confers tumor suppressive effect on therapy-resistant breast tumor cells

**DOI:** 10.1186/bcr2929

**Published:** 2011-08-11

**Authors:** Binoj C Nair, Sreeram Vallabhaneni, Rajeshwar R Tekmal, Ratna K Vadlamudi

**Affiliations:** 1 Department of Obstetrics and Gynecology and CTRC at UT Health Science Center,7703 Floyd curl drive, San Antonio, Texas, 78229, USA; 2 Department of Molecular Medicine, UT Health Science Center, 7703 Floyd curl drive, San Antonio, Texas, 78229, USA

## Abstract

**Introduction:**

Current clinical strategies for treating hormonal breast cancer involve the use of anti-estrogens that block estrogen receptor (ER)α functions and aromatase inhibitors that decrease local and systemic estrogen production. Both of these strategies improve outcomes for ERα-positive breast cancer patients, however, development of therapy resistance remains a major clinical problem. Divergent molecular pathways have been described for this resistant phenotype and interestingly, the majority of downstream events in these resistance pathways converge upon the modulation of cell cycle regulatory proteins including aberrant activation of cyclin dependent kinase 2 (CDK2). In this study, we examined whether the CDK inhibitor roscovitine confers a tumor suppressive effect on therapy-resistant breast epithelial cells.

**Methods:**

Using various *in vitro *and *in vivo *assays, we tested the effect of roscovitine on three hormonal therapy-resistant model cells: (a) MCF-7-TamR (acquired tamoxifen resistance model); (b) MCF-7-LTLTca (acquired letrozole resistance model); and (c) MCF-7-HER2 that exhibit tamoxifen resistance (ER-growth factor signaling cross talk model).

**Results:**

Hormonal therapy-resistant cells exhibited aberrant activation of the CDK2 pathway. Roscovitine at a dose of 20 μM significantly inhibited the cell proliferation rate and foci formation potential of all three therapy-resistant cells. The drug treatment substantially increased the proportion of cells in G2/M cell cycle phase with decreased CDK2 activity and promoted low cyclin D1 levels. Interestingly, roscovitine also preferentially down regulated the ERα isoform and ER-coregulators including AIB1 and PELP1. Results from xenograft studies further showed that roscovitine can attenuate growth of therapy-resistant tumors *in vivo*.

**Conclusions:**

Roscovitine can reduce cell proliferation and survival of hormone therapy-resistant breast cancer cells. Our results support the emerging concept that inhibition of CDK2 activity has the potential to abrogate growth of hormonal therapy-resistant cells.

## Introduction

The steroid hormone estradiol (E2) plays an important role in the initiation and progression of breast cancer. Various biological effects of E2 are mediated through its binding to distinct estrogen receptors (ER), ERα and ERβ, that differ both functionally and structurally [1,2]. About 70% of breast cancer patients are ERα positive at the time of presentation [3]. Upon E2 binding, ERα mediates regulation of target gene transcription and cell cycle progression via recruitment of co-regulators [2]. Emerging evidence suggests that ERβ decreases cell proliferation, that breast tumors express low levels of ERβ and that the ratio between ERα and ERβ is the driving force for tumor cell proliferation [4]. Current endocrine therapy for ER-positive breast cancer involves modulating the ER-pathway using either anti-estrogens (AEs) or aromatase inhibitors (AIs). Despite the positive effects, *de novo *and/or acquired resistance to endocrine therapies frequently occur [5]. Although mechanisms for hormonal therapy resistance remains elusive, emerging data suggest that ER cross talk with human epidermal growth factor receptor 2 (HER2)-, Src-, and AKT-pathways, that alterations in the levels of ER subtypes, and that deregulation of co-regulator are major causes of resistance [6-8].

Interestingly, most downstream events in these resistance signaling pathways converge upon modulation of cell cycle regulatory proteins; the most conspicuous of which is the upregulation of cyclins E and A, along with activation of cyclin dependent kinase 2 (CDK2) [9,10]. The cell cycle machinery is also a prime target for both estrogen and AEs to enhance cell cycle progression or to induce cell cycle arrest, respectively [11]. CDK2 is known to aid in cancer cell proliferation by modulating E2F-pRB pathway [12] and is also shown to enhance ligand-independent activation of ERα [13,14]. The expression of RB/E2F target genes, which is tightly controlled by CDK2 activity, is often deregulated and associated with worse prognosis for tamoxifen-treated breast cancer patients [15]. Collectively, these emerging studies strongly support the concept that CDK2 activity is a critical component for the generation of a hormone therapy-resistant phenotype and that blocking of CDK2 activity may be useful as a therapeutic strategy for therapy-resistant patients.

(R)-stereoisomer of roscovitine (Seliciclib or CYC202) is one of the extensively studied CDK inhibitors, both *in vitro *and *in vivo *[16]. Roscovitine is the first, selective, orally bioavailable inhibitor of CDKs to enter clinical trials [17] and is currently in phase II trials for B-cell malignancies, and lung cancer [18]. It predominantly inhibits CDK2, and has a very short half-life with no known active metabolites [19]. Earlier studies have shown that roscovitine promotes accumulation of breast tumor cells in G2/M phase [20,21], potentiates the anti-tumor effects of doxorubicin on breast cancer cells [22], and has a synergistic antitumor effect with irradiation in a breast cancer xenograft model [23]. Although these studies suggested that roscovitine may have therapeutic utility in the management of hormonal therapy-sensitive breast cancer cells, the utility of roscovitine to suppress endocrine therapy-resistant breast cancer cells has not been explored.

In this study, we evaluated the tumor-suppressive effect of roscovitine using three different breast cancer models that exhibit resistance to hormonal therapy. Our findings using *in vitro *and *in vivo *xenograft assays demonstrate that roscovitine has the potential to reduce growth of all three therapy-resistant cells. Mechanistic studies revealed that roscovitine actions involve both blocking of CDK functions as well as down-regulation of ERα. As roscovitine is currently in clinical trials, our findings may have a high translational potential, suggesting that roscovitine represents a novel therapeutic drug for treating therapy-resistant tumors.

## Materials and methods

### Model cells and reagents

MCF7 cells were purchased from American-type culture collection (ATCC, Manassas, VA, USA), endocrine-resistant model cells MCF7-HER2 [24], MCF7-tamoxifen resistant (TamR) [24] and long-term letrozole treated MCF7ca (MCF7LTLTca) [25] were described. MCF7-LTLTca and MCF7-TamR cells were cultured in phenol red free-RPMI medium containing 5% dextran charcoal-treated serum with either 1 μmol/L of letrozole or 1 μmol/L tamoxifen, respectively. The roscovitine that was used for the *in vitro *studies was purchased from Calbiochem (San Diego, CA, USA) and that was used for the *in vivo *studies was purchased from LC Laboratories (Woburn, MA, USA). Antibodies for phospho-Rb, phospho-CDK2, and AIB1 were purchased from Cell Signaling (Beverly, MA, USA). The antibody for PELP1 was purchased from Bethyl Laboratories (Montgomery, TX, USA). ERα was purchased from Santa Cruz Biotechnology (Santa Cruz, CA, USA) and Thermo Fisher Scientific (Rockford, IL, USA). terminal deoxynucleotidyl transferase dUTP nick end labeling (TUNEL) kit for apoptosis detection was purchased from (Roche, Mannheim, Germany) and Proliferating Cell Nuclear Antigen (PCNA) antibody was purchased from Vector Lab (Burlingame, CA, USA).

### Cell lysis and western blot analysis

Cells were washed with ice cold 1 × PBS and lysis was conducted using a modified RIPA buffer (150 mM NaCl, 50 mM Tris-HCl, 50 mM NaF, 5 mM EDTA, 0.5% (wt/vol) sodium deoxycholate and 1% Triton X-100) containing phosphatase and protease inhibitors. Lysates were run on either 7% or 8% SDS-PAGE. Western blot analysis was performed with phospho- and total antibodies. For protein degradation analysis, model cells were pretreated with MG132 (5 μM) for one hour prior to treatment with 20 μM roscovitine.

### Cell proliferation assay

The cell proliferation rate was measured in a 96-well, flat, clear-bottom, opaque-wall microplates using Cell Titer-Glo Luminescent Cell Viability Assay (Promega, Madison, WI, USA). Model cells (2 × 10^3^) were plated in each well and cultured for 24 hours before treatment with or without various doses of roscovitine for another 72 hours. Total ATP content as an estimate of total number of viable cells was measured by luminescence-based assay using automatic Fluoroskan Luminometer (Thermo Scientific Waltham, MA, USA). Roscovitine was dissolved in dimethyl sulfoxide (DMSO) as 2.8 mM stock solution and stored as aliquots at -20°C. All roscovitine treatments were performed in phenol red-free medium as described [26].

### Clonogenic assay

Model cells were plated in six-well plates at a density of 1 × 10^3 ^cells per well in triplicate and treated with or without 10 μM roscovitine for seven days. After three weeks, cells were fixed in cold-methanol, and stained with 0.5% crystal violet. Image acquisition was conducted using a digital camera.

### Flow cytometry

Model cells were plated in 100 mm plates and treated with or without 20 μM roscovitine for 24 hours. Cells were trypsinized and harvested in 1 × PBS, followed by fixation in ice cold 70% ethanol. Staining was done with a mixture of 50 μg/mL propidium iodide and 50 μg/mL RNase A. Cell cycle status was quantified by using a FACS-Calibur flow cytometer.

### Quantitative RT-PCR analysis

Model cells were plated in 100 mm plates and treated with or without roscovitine (20 to 30 μM) for 24 hours. Cells were harvested with Trizol Reagent (Invitrogen, Carlsbad, CA, USA) and total RNA was isolated according to the manufacturer's instructions. cDNA synthesis was done using Superscript III RT-PCR kit (Invitrogen, Carlsbad, CA, USA). Real-time PCR was done using a Cepheid Smartcycler II (Sunnyvale, CA, USA) with specific real-time PCR primers for the ERα (ESR1-F: 5'AGAGGGAAAGTAGGGCAGAA 3' and ESR1-R: 5'TGGGAAATGAAGAAGAGCTG 3'). Results were normalized to actin transcript levels and the difference in fold expression was calculated using delta-delta-CT method.

### *In vivo *xenograft assays

All animal experiments were performed after obtaining University of Texas Health Sciences Centre at San Antonio (UTHSCSA) institutional animal care committee (IACUC) approval and the animals were housed in accordance with UTHSCSA institution's protocol for animal experiments. Model cells (5 × 10^6^) cells mixed with an equal volume of Matrigel were implanted subcutaneously into the flanks of six to seven-week-old female nude mice as described [27]. For MCF7-HER2 xenografts, nude mice were also subcutaneously implanted with estrogen pellets (Innovative Research, Sarasota, FL, USA) [28]; whereas nude mice with MCF7-TamR and MCF7-LTLTca xenografts were given subcutaneous injection of tamoxifen (100 μg/day/mice) or Δ4-androstenedione (100 μg/day/mice), respectively, as described [29]. Roscovitine treatment was initiated after three weeks of inoculation when tumors reached measurable size. Roscovitine suspension was prepared in 50 mM HCl solution as described earlier [30] and administered orally at a dose of 100 mg/kg body weight (three times a day) for 10 consecutive days (total 30 doses). Tumor volumes were measured with a vernier caliper at weekly intervals. After the 25^th ^day, the mice were euthanized, and the tumors were removed, weighed and processed for immunohistochemistry (IHC) staining. Tumor volume calculation was performed using modified ellipsoidal formula: *tumor volume *= 1/2(*L *× *W*^2^), where L is the longitudinal diameter and W is the transverse diameter [27]. Body weight was measured at weekly intervals to rule out the drug toxicity. Student t-test was used to assess the statistical difference between control and roscovitine-treated groups. The level of significance was set at *P *< 0.05.

### Immunohistochemistry and TUNEL assay

IHC analysis was performed as described [27]. The dilution of the PCNA antibody used for IHC was 1 in 100. After washing with 1 × PBST, slides were further incubated in DAKO universal secondary LINK solution for 30 minutes and horseradish peroxidase-labeled streptavidin for 20 minutes at room temperature. Successful staining was visualized using the DAB substrate (Vector Lab, Burlingame, CA, USA), followed by counterstaining using Mayer's hematoxylin (Vector Lab, Burlingame, CA, USA). TUNEL staining was performed following manufacturer's instructions (Roche, Indianpolis, IN).

## Results

### Roscovitine suppresses proliferation and survival of therapy-resistant cells

**Figure 1:**
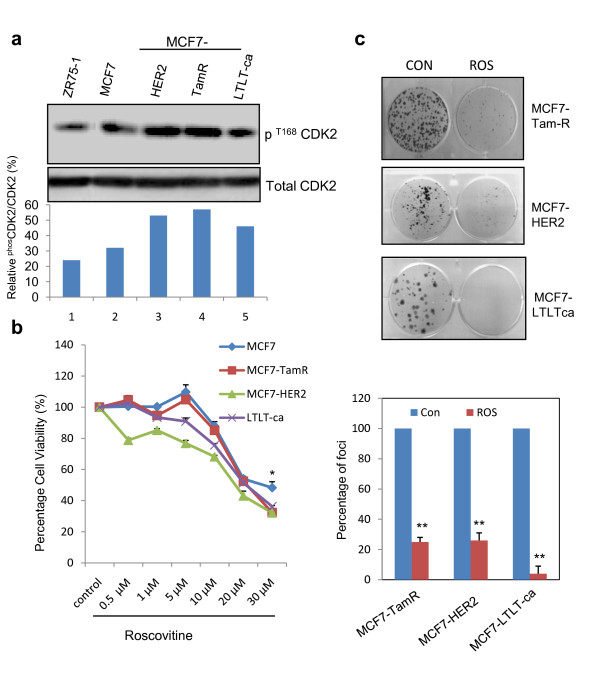
**Effect of roscovitine on cell proliferation and survival of endocrine-resistant cancer cells**. **(a) **Total cellular lysates from control and therapy-resistant model cells were subjected to western blotting using phosphor cell cycle dependent kinase (CDK) 2 antibody. Graph shows densitometry measurements of phosCDK2 protein bands relative to total CDK2 in each cell line. **(b) **Model cells were treated with or without roscovitine and cell proliferation was determined at indicated time points by using the Cell Titer Glo assay. **(c) **Model cells were treated with or without roscovitine and clonogenic survival was determined. Representative figures and quantitative analysis of survival was shown. Statistical significance was determined by student's t-test. P, *P *value; * *P *< 0.05; ** *P *< 0.01. Con, control; Phos, phospho; ROS, roscovitine.

To examine whether deregulation of CDK2 signaling occurs in therapy-resistant cells, we initially determined the status of CDK2 activation in therapy-resistant model cells that exhibit therapy resistance to AE (MCF7-HER2, and MCF7-TamR) and AI (MCF7LTLTca). CDK2 activation was determined by measuring the levels of phospho-CDK2 (T160) by using western blot analysis. Phospho-CDK2 levels were enhanced in all three therapy-resistant model cells but not in the hormone-sensitive breast cancer cells such as MCF7 and ZR-75-1 (Figure 1a). We then tested whether roscovitine suppresses the growth of therapy-resistant cells by exposing them to increasing concentrations of roscovitine and determined their rate of proliferation using a luminescence-based cell proliferation assay. Hormonal therapy-sensitive MCF7 cells were used as a positive control and these cells had a dose-dependent reduction in cell proliferation. Interestingly, all the three endocrine-resistant cells also had concomitant reductions in cell proliferation upon roscovitine treatment (Figure 1b) with 50% or more reduction in cell proliferation (IC_50_) at a dose range of 20 to 30 μM. Compared with MCF7 cells, resistant cells were more sensitive to 30 μM roscovitine. We next examined whether, roscovitine affects clonogenic survival of endocrine-resistant cells by using an *in vitro *clonogenic survival assay. We treated therapy-resistant models cells with 20 μM roscovitine for seven days and measured their clonogenic ability after three weeks (Figure 1c*upper panel*). Compared with untreated cells, MCF7-TamR and MCF7-HER2 cells had about a 75% reduction in the colony formation potential and LTLTca cells had about 4% colony formation (Figure 1c*lower panel)*. Overall, these results suggest that roscovitine has the potential to suppress the proliferation and survival potential of endocrine-resistant cells.

### Roscovitine arrests the cell cycle in endocrine-resistant cells

**Figure 2:**
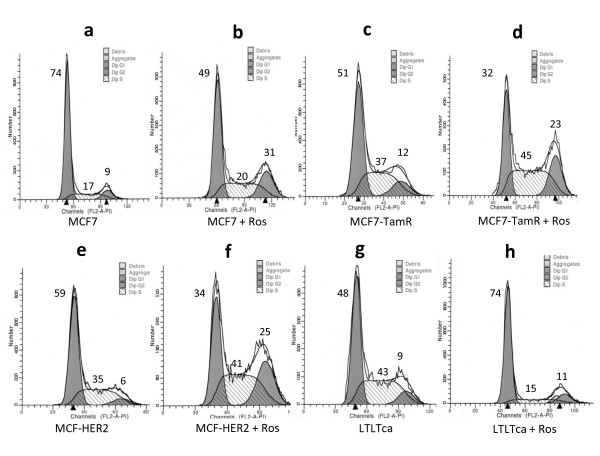
**Roscovitine modulates cell cycle status of endocrine-resistant cancer cells**. **(a, c, e, g) **Control cells and/or **(b, d, f, h) **cells that were treated with roscovitine were subjected to flow cytometry. The percentage of cells in each cell cycle phase is depicted on the top of corresponding G1, S, and G2/M peaks. ROS, roscovitine.

Previous studies found that roscovitine has the potential to perturb cell cycle progression in various cell lines [26]. To evaluate whether roscovitine promotes cell cycle arrest in endocrine-resistant cells, we treated three resistant model cells with 20 μM roscovitine for 24 hours. Compared with their untreated cells, roscovitine-treated MCF7, MCF7-TamR, and MCF7-HER2 cells had substantial more cells containing 4N DNA content (G2/M phase) and concurrently less cells in the G1 phase (Figure 2a to 2f). However, 74% of roscovitine-treated LTLTca cells accumulated in the G1 phase and 48% of the untreated LTLTca cells accumulated in the G1 phase (Figure 2g and 2h). Collectively, these results suggest that roscovitine has potential to block cell cycle progression of endocrine therapy-resistant cells and preferentially arrest them at the G2/M or G1 phase of cell cycle.

### Roscovitine down regulates key cell cycle regulators and ERα levels

**Figure 3:**
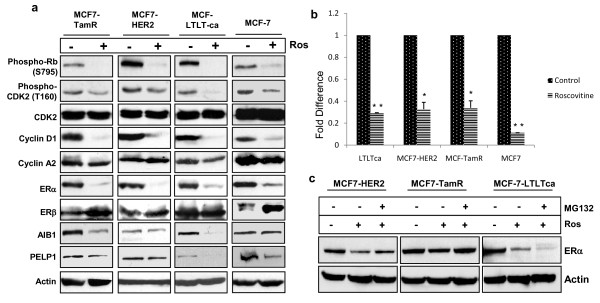
**Roscovitine treatment suppresses expression of key regulators of cell cycle and the ER α-signaling axis**. **(a) **The model cells MCF7, MCF7-TamR, MCF7-HER2, and MCF7-LTLTca were treated with roscovitine and the status of cell cycle regulators and the estrogen receptor (ERα) signaling proteins was analyzed by western blotting. **(b) **MCF7, MCF7-Tam, MCF7**-**LTLTca, and MCF7-HER2 were treated with roscovitine and the levels of ERα transcripts were determined by quantitative RT-PCR. **(c) **Model cells were pre-treated with the proteosomal inhibitor MG132 and then subjected to roscovitine treatment. ERα status was determined by western blotting of total lysates. Actin was used as loading control. Con, control; ROS, roscovitine.

As roscovitine induced cell cycle arrest in endocrine-resistant cells, we next examined the expression of key cell cycle regulators in roscovitine-treated endocrine-resistant cells. As expected, roscovitine treatment reduced CDK2 activity as detected by less CDK2 threonine 160 phosphorylation in all three resistant cells and also in MCF7 cells (Figure 3a). Further, the level of phospho-Rb, a well-known substrate of CDK2, was reduced after roscovitine treatment, confirming the down regulation of CDK2 axis in roscovitine-treated cells (Figure 3a). Roscovitine-treated endocrine-resistant cells also had reductions in the levels of cyclin D1 with no or little change in cyclin A2. Interestingly, roscovitine treatment specifically down regulated the ERα isoform expression but had little effect on ERβ expression (Figure 3a). Furthermore, roscovitine down regulated co-activators of ERα such as AIB1 and PELP1, which also play predominant roles in hormonal cell cycle progression and resistance [31,32] (Figure 3a). As ERα expression is regulated at both transcriptional and translational levels, we examined whether the down regulation of ERα is due to transcriptional or post-translational effects of roscovitine. We found significantly less ERα mRNA levels in roscovitine-treated MCF7 and therapy-resistant cells than in their untreated counterparts (Figure 3b). Further, treatment of cells with proteosomal inhibitor MG132 was also able to partially rescue the degradation of ERα with roscovitine treatment (Figure 3c). These results suggest that roscovitine can block both CDK2 signaling axis as well as down regulate specific components of ERα signaling axis.

### Therapeutic efficacy of roscovitine on xenografts generated from endocrine-resistant model cells

**Figure 4:**
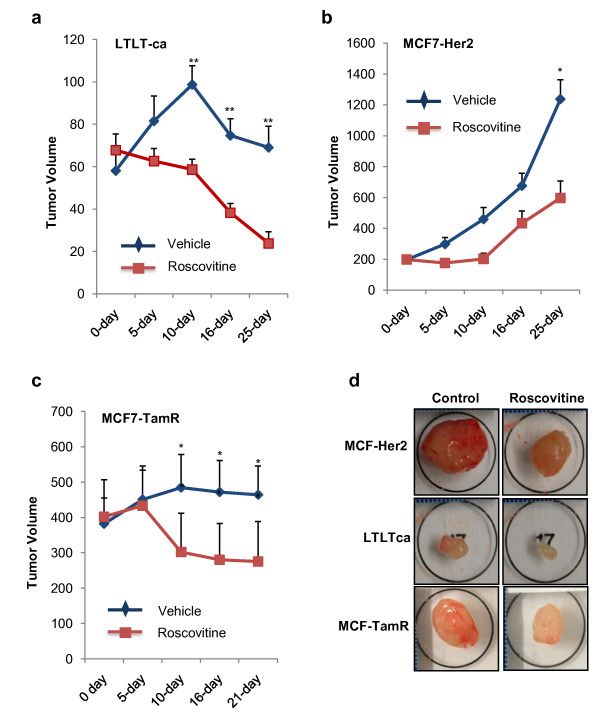
**Roscovitine exerts a tumor suppressive effect on endocrine-resistant breast cancer xenografts**. Nude mice were subcutaneously injected with the endocrine-resistant model cells **(a) **MCF7-LTLTca (*n *= 8), **(b) **MCF7-HER2 (*n *= 8), and **(c) **MCF7-TamR (*n *= 6). After three weeks, roscovitine treatment was given for 10 consecutive days and tumor volumes were recorded on the 5^th^, 10^th^, 16^th^, and 25^th ^day after roscovitine treatment. P, *P *value; ** P < 0.01, * *P *< 0.05. **(d) **Representative images of tumors depicting the size of tumors derived from both control and roscovitine-treated groups.

**Figure 5:**
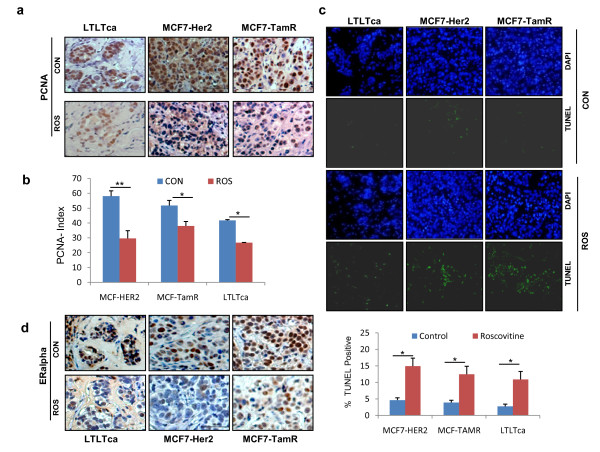
**Roscovitine treatment reduces proliferation and increases apoptosis in endocrine-resistant xenografts**. **(a) **Immunohistochemical analysis of Proliferating Cell Nuclear Antigen (PCNA) on tumors treated with or without roscovitine. **(b) **Quantitation of PCNA staining using the PCNA index. P, *P *value; ** P < 0.01, * *P *< 0.05. **(c) **TUNEL staining for apoptosis in control and roscovitine-treated tumors. Representative images are depicted (upper panel). Terminal deoxynucleotidyl transferase dUTP nick end labeling (TUNEL) labeling was quantified as the mean TUNEL labeling percentage based on at least three randomly selected high-power microscope fields per group (lower panel). P, *P *value; ** P < 0.01, * *P *< 0.05. **(d) **Immunohistochemical analysis for estrogen receptor (ERα) status was performed in groups with or without roscovitine treatment. Con, control; ROS, roscovitine.

To examine whether roscovitine inhibits growth of therapy-resistant cells *in vivo*, we used a nude mice-based xenograft assay. After three weeks of implantation and when tumors reached measurable size, roscovitine or vehicle was given orally at a dose of 100 mg/kg/mice/day, three times a day for 10 consecutive days. Tumor volume was measured every week and after 25 days the last treatment, mice were euthanized. For all three models, roscovitine-treated mice had significantly smaller tumor volumes (Figure 4a to 4c) and smaller tumor sizes (Figure 4d). No toxicities were observed in behavioral changes, such as eating habits and mobility, in animals treated with roscovitine and mouse weights were not significantly different between control and roscovitine-treated groups (data not shown). IHC analysis for PCNA, a well-established proliferation marker, revealed less PCNA staining in all the roscovitine-treated tumors (Figure 5a). A reduction in the PCNA index with roscovitine treatment was more significant in MCF7LTLTca xenografts than in MCF7-TamR and MCF7-HER2 xenografts (Figure 5b). Furthermore, compared with untreated xenografts, roscovitine-treated xenografts had greater levels of apoptosis when assayed using TUNEL staining (Figure 5c). IHC analysis using ERα-specific antibody revealed less ERα staining in roscovitine-treated cells than in the untreated cells (Figure 5d). Overall these results suggest that roscovitine can suppress cell proliferation of therapy-resistant cells and lead to apoptosis.

## Discussion

Breast cancer is the most common cancer among women in the USA and patients with ERα-positive tumors greatly benefit from existing hormonal therapies using AEs and AIs. [33-36]. However, many patients exhibit *de novo *or acquired resistance to hormonal therapies. This resistance represents a major clinical problem. Emerging findings suggest that deregulation of cell cycle components such as CDK2 axis has the potential to contribute both to cell cycle progression and to endocrine resistance [11]. In this study, we explored the hypothesis that targeting the CDK2 axis using roscovitine will have therapeutic benefit. Using three model cells that exhibit endocrine therapy resistance, we found that (1) therapy-resistant cells exhibit greater CDK2 activation; (2) roscovitine treatment decreased CDK2 activity and promoted G2/M or G1 accumulation of resistant cells; (3) roscovitine treatment significantly reduced proliferation of the resistant cells; (4) roscovitine preferentially down regulated the ERα isoform and its associated signaling components; and (5) preclinical xenograft-based studies found roscovitine had a therapeutic efficacy by attenuating the growth of resistant tumors. Collectively, these results suggest that CDK2 signaling confers a growth advantage to resistant cells and that roscovitine treatment represents a feasible strategy for therapeutic targeting of hormonal therapy resistance.

Tumor cells exhibit oncogenic addiction. Recent studies suggest that interphase CDKs (CDK4, CDK2) are only essential for proliferation of tumor cells and selective CDK inhibition may provide therapeutic benefit [37]. Previous studies demonstrated the potential of roscovitine to abrogate cell proliferation and to induce cell cycle arrest in both ER-positive cells [21,26,38] and ER-negative cells [39]. In this study, we demonstrated that roscovitine exhibited a profound growth inhibitory effect on hormone-resistant model cells. Roscovitine treatment promoted G2/M arrest in MCF7-TamR and MCF7-HER2 cells and arrested in LTLTca cells in the G1 phase. These findings are in agreement with the findings from published studies that concluded roscovitine has the potential to arrest cell cycle predominantly at G2/M phase and occasionally in the G1 phase [22,38,40,41]. The differential effect of roscovitine on cell cycle status in LTLTca compared with TamR and HER2 cells is currently unknown, but we speculate the difference may lie in differential activation of cell cycle checkpoints in these model cells. Utility of roscovitine against letrozole-resistant breast cancer cells have recently been demonstrated [42] and our data strongly corroborate this study.

Activation of the CDK2 axis is considered a vital end point of various molecular pathways leading to hormone-therapy resistance [11]. Cyclin E, an activator of CDK2, when ectopically over-expressed is able to abrogate the anti-proliferative actions of tamoxifen on breast cancer cells [9] and is also shown to be a good indicator for endocrine-therapy failure [43,44]. Recent studies showed that cyclin E can undergo proteolytic cleavage to create low molecular weight (LMW) cyclin E variants [45] and these LMW cyclin E variants lacking the amino-terminus could significantly augment the tamoxifen therapy resistance by activating CDK2 functions [46]. LMW cyclin E is also reported to abrogate the anti-proliferative activity of the AI letrozole [42]. Overexpression of cyclin A in breast cancer also significantly correlates with poor outcome in tamoxifen-treated patients [47]. Down regulation of CDK inhibitors (p21 and p27) have been implicated in the development of hormone therapy resistance [48,49]. In this study, we show that roscovitine, a potent inhibitor of CDK2, can curb the growth of therapy-resistant breast cancer cells and to down regulate expression of ERα. As deregulation of the cell cycle machinery and ER signaling both contribute to hormonal therapy resistance, a roscovitine treatment regime that suppresses both these pathways could serve as a double-edged sword to interfere with the hormonal therapy-resistant mechanisms.

Cell cycle regulators cyclins and CDKs are the key proteins that aid in cell proliferation by modulating the E2F-RB pathway [12]. Earlier studies that showed functional ablation of retinoblastoma protein (pRb) leads to activation of CDK2 in breast cancer [15,50] and concordantly, that deregulation of E2F transcription factor target genes associated with poor prognosis [51]. Our results suggested that roscovitine can reduce phosphorylation of Thr160 in CDK2 (a marker of CDK2 activation), reduce phosphorylation of pRb (a well-known substrate of CDK2) at Ser795, and reduce levels of cyclin D1 in therapy-resistant cells. The ability of roscovitine to reduce pRb phosphorylation and to alter the status of cyclin D1, suggest that roscovitine therapy may have therapeutic benefit on clinical cases with CDK2 activation and deregulation of E2F functions.

Cross talk between the cell cycle machinery and ER pathways has been well documented in the context of endocrine resistance [11]. CDKs are known to potentiate ER functions in AE-resistant cells [13] and CDK2 activity significantly correlates with poorer five-year relapse-free survival in patients [52]. Interestingly, in our study, *in vitro *roscovitine treatment reduced the expression of both ERα and ER co-regulators such as AIB1 and PELP1, which are commonly implicated in therapy resistance [31,32]. Our findings suggest that the reduction in ERα levels in the resistant cells is due to both transcriptional and post-translational effects by roscovitine. Our data corroborate data from a recent study that demonstrated a reduction in ERα levels in MCF7 cells after roscovitine treatment, which possibly occurred through the inhibition of CDK7 [53]. As many endocrine therapy-resistant cells retain expression and functionality of ERα, the ability of roscovitine to down regulate the ERα axis, may also have contributed to its ability to curb the progression of the resistant cells.

(*R*)-roscovitine is currently in early clinical trials to examine its effects on treating non-small cell lung cancer and advanced solid tumors [12]. The oral bioavailability of (*R*)-roscovitine is a great advantage for its possible future clinical use. In this study, we tested efficacy of roscovitine *in vivo *by using a xenograft transplantation assay. Our data suggest that roscovitine has strong tumor suppressive effects on endocrine-resistant xenograft tumors. To avoid possible side effects of roscovitine, we chose a rather moderate dose (100 mg/kg) of the drug; a few other studies used 400 mg/kg roscovitine [22]. IHC analysis of tumors revealed a significant decrease in proliferation along with an increase in apoptosis. Our data corroborate data from other studies that demonstrated the apoptotic potential of roscovitine [39,54] and in turn suggest that multiple pathways are responsible for roscovitine-induced tumor suppressive effects.

## Conclusions

Our results support the concept that inhibition of CDK2 activity has the potential to abrogate growth of hormonal therapy-resistant cells. Further, the results from this study provide strong *in vitro *and *in vivo *evidence that roscovitine confers a tumor-suppressive effect on endocrine therapy-resistant breast tumor cells by inhibiting CDK functions and altering expression of ERα and ER-coregulators, and by promoting apoptosis. Future studies are needed to examine whether roscovitine treatment can be used to treat advanced and therapy-resistant breast cancer and whether roscovitine treatment can complement the existing therapies.

## Abbreviations

AE: anti-estrogen; AI: aromatase inhibitor; CDK: cyclin dependent kinase; E2: estradiol; ER: estrogen receptor; HER2: human epidermal growth factor receptor 2; IHC: immunohistochemistry; LMW: low molecular weight; LTLTca: long-term letrozole treated MCF7ca; PBS: phosphate-buffered saline; PCNA: Proliferating Cell Nuclear Antigen; pRb: retinoblastoma protein; TamR: tamoxifen resistant; TUNEL: terminal deoxynucleotidyl transferase dUTP nick end labeling.

## Competing interests

The authors declare that they have no competing interests.

## Authors' contributions

BCN performed the majority of the *in vitro *experiments, xenograft studies, coordinated with all the team members and drafted the manuscript. SV performed some of the western blot analysis and a couple of IHC assays. RT participated in the design of the study and performed the analysis of the IHC data. RV conceived the study, and participated in the design of the project and preparation of final manuscript. All authors read and approved the final manuscript for publication.
